# Culex flavivirus infection in a *Culex pipiens* mosquito colony and its effects on vector competence for Rift Valley fever phlebovirus

**DOI:** 10.1186/s13071-018-2887-4

**Published:** 2018-05-23

**Authors:** Sandra Talavera, Lotty Birnberg, Ana I. Nuñez, Francesc Muñoz-Muñoz, Ana Vázquez, Núria Busquets

**Affiliations:** 1 IRTA, Centre de Recerca en Sanitat Animal (CReSA, IRTA-UAB), Campus de la Universitat Autònoma de Barcelona, 08193 Bellaterra, Spain; 2 Departament de Biologia Animal, de Biologia Vegetal i d’Ecologia, Universitat Autònoma de Barcelona, 08193 Bellaterra, Spain; 3 National Centre for Microbiology, Instituto de Salud Carlos III, Ctra. Majadahonda-Pozuelo, km. 2, 28220 Majadahonda, Madrid, Spain; 4 CIBER de Epidemiología y Salud Pública. CIBERESP, Instituto de Salud Carlos III, Ctra. Majadahonda-Pozuelo, km. 2, 28220 Majadahonda, Madrid, Spain

**Keywords:** Rift Valley fever phlebovirus, *Culex pipiens*, Culex flavivirus, Transmission, Vector competence

## Abstract

**Background:**

Rift Valley fever is a mosquito-borne zoonotic disease that affects domestic ruminants and humans. Culex flavivirus is an insect-specific flavivirus that naturally exists in field mosquito populations. The influence of Culex flavivirus on Rift Valley fever phlebovirus (RVFV) vector competence of *Culex pipiens* has not been investigated.

**Methods:**

Culex flavivirus infection in a *Cx. pipiens* colony was studied by Culex flavivirus oral feeding and intrathoracical inoculation. Similarly, vector competence of *Cx. pipiens* infected with Culex flavivirus was evaluated for RVFV. Infection, dissemination, transmission rates and transmission efficiency of Culex flavivirus-infected and non-infected *Cx. pipiens* artificially fed with RVFV infected blood were assessed.

**Results:**

Culex flavivirus was able to infect *Cx. pipiens* after intrathoracically inoculation in *Cx. pipiens* mosquitos but not after Culex flavivirus oral feeding. Culex flavivirus did not affect RVFV infection, dissemination and transmission in *Cx. pipiens* mosquitoes. RVFV could be detected from saliva of both the Culex flavivirus-positive and negative *Cx. pipiens* females without significant differences. Moreover, RVFV did not interfere with the Culex flavivirus infection in *Cx. pipiens* mosquitoes.

**Conclusions:**

Culex flavivirus infected and non-infected *Cx. pipiens* transmit RVFV. Culex flavivirus existing in field-collected *Cx. pipiens* populations does not affect their vector competence for RVFV. Culex flavivirus may not be an efficient tool for RVFV control in mosquitoes.

## Background

Culex flavivirus (CxFV) belongs to the genus *Flavivirus* (family *Flaviviridae*). The majority of viruses within this genus are transmitted horizontally between vertebrate hosts and hematophagous arthropods. However, some flaviviruses are considered to be vertebrate-specific while other group of viruses of this genus are insect-specific (ISFV) [1–3]. Circulation of ISFVs in natural mosquito populations is likely maintained by vertical transmission [4, 5]. In Europe, several species of ISFV have been detected in field mosquitoes from Italy, Portugal, Spain, the United Kingdom, the Czech Republic and Greece [6–10]. Sequences related to those viruses have been detected worldwide [11–14]. ISF RNA has also been detected in sand flies (family *Psychodidae*) in Algeria [2], Spain [3] and Portugal (GenBank: HM563684). Previous field studies in Spain suggested the existence of a large number of ISF [3, 15, 16], though not completely characterized phylogenetically [10]. The circulation of ISFV in nature raises concerns regarding possible interactions with arthropod-borne flaviviruses [17] and even other arboviruses in vector populations. Co-infection studies with mosquito-borne flaviviruses (MBFV) and ISFV have been performed in order to gain a better understanding of any factor that could alter vector competence of mosquitoes for MBFV in both enzootic and epizootic transmission cycles [18]. Three studies were carried out to directly address potential co-infection exclusion effect between CxFV and other flaviviruses such as West Nile virus (WNV) [18–20]. However, no co-infection studies with other pathogenic viruses belonging to other genera have been performed, such as Rift Valley fever phlebovirus (RVFV).

Rift Valley fever (RVF) is a mosquito-borne zoonotic disease caused by RVFV (genus *Phlebovirus*, family *Phenuiviridae*). RVFV is transmitted by mosquito bites to a large number of hosts, both domestic and wild ruminants [21]. Described for the first time in 1931 in Kenya [22], RVFV has continuously caused outbreaks in animals and humans in several African countries [23]. In 2000, RVFV was first reported outside of Africa, i.e. in Saudi Arabia and Yemen [24], linking to the likelihood of a potential introduction of RVFV in Europe. The risk of RVFV introduction in Europe has been recently evaluated [25–28]. Results of a multiple criteria decision-making model study of key factors for RVF in Spain identified areas with high suitability for RVF outbreak occurrence in each month of the year [28]. Moreover, a previous study has shown that a *Culex pipiens* mosquito colony from Spain is able to transmit this virus [29]. Species of the genera *Aedes* and *Culex* are considered main vectors of RVFV [30]. *Culex pipiens* complex is considered as an efficient RVFV vector [31] including *Cx. pipiens* and *Cx. quinquefasciatus* which are ubiquitous mosquitoes in temperate and tropical regions, respectively [32].

It is relevant to understand ISF dynamics and their role in their mosquito hosts as potential control tool for vector-borne pathogens. To this end, the objectives of the present study were to evaluate (i) the CxFV infection in a *Cx. pipiens* colony by oral feeding and intrathoracic inoculation and (ii) the role in vector competence of CxFV for RVFV infection, dissemination and transmission by *Cx. pipiens*. All experiments were performed simulating environmental conditions of the season with high vector density and high suitability for RVF outbreak occurrence in the distribution area of the tested mosquito population.

## Methods

### Mosquito populations

One mosquito population of *Cx. pipiens pipiens* and *molestus* hybrid form from Gavà (2012), Catalonia (northeastern Spain) was used. Molecular characterization of the *Cx. pipiens* forms was performed for each individual involved in the RVFV vector competence assay as previously described [33]. The *Cx. pipiens* colony was reared in laboratory under environmental conditions: temperature, 26 °C:22 °C (day:night); relative humidity (RH) of 80%; and a 14:10 h (L:D) photoperiod including two crepuscular cycles of 30 min to simulate dawn and dusk.

Before vector competence assays, the mosquito colony was tested for the presence of viruses, as described previously [29], to exclude other viral infections (species of *Flavivirus*, *Alphavirus* and *Phlebovirus*). In the last decade, other novel insect-specific viruses have been detected in field mosquitoes belonging to several families such us *Bunyaviridae*, *Mesoniviridae*, *Reoviridae*, *Rhabdoviridae*, *Togaviridae* and the newly recognized taxon of *Negeviruses* [34]. Prior to vector competence assays, the colony was also tested for the presence of these viruses using generic RT-nested-PCR (unpublished) and *Wolbachia* spp. by PCR [35]. The mosquito colony was found to be *Wolbachia* spp.-positive and negative for *Flavivirus*, *Alphavirus*, *Phlebovirus*, *Bunyaviridae*, *Mesoniviridae*, *Reoviridae*, *Rhabdoviridae*, *Togaviridae* and *Negeviruses* (data not shown).

### Virus strains

The CxFV strain was detected in field-collected *Culex pipiens* mosquitoes captured in Huelva, Spain, in 2006, and isolated in C6/36 cells. To propagate the virus, C6/36 cells were incubated for 6–7 days (28 °C, 5% CO_2_) and viral particles were observed by electronic microscopy. As cytopathic effect was not observed, CxFV replication was detected in the supernatant using a modified real time RT-PCR [19] (see below). A monolayer of C6/36 cells was used to titrate CxFV. Briefly, eight wells were infected for each ten-fold dilution. Twenty microliters of inoculum and 150 μl of minimum essential medium (Life Technologies, Carlsbad, CA, USA) supplemented with 2% FBS (EuroClone SpA, Pero, Italy), 2 mM L-glutamine, non-essential amino acids, 1000 U/ml of penicillin, 10 mg/ml of streptomycin and 500 U/ml of nystatin (all from Sigma-Aldrich, St. Louis, MO, USA), were added into each well as post-infection medium. Plates were incubated at 28 °C and 5% of CO_2_ for 7 days. Calculation of the viral titer was performed by virus detection in each well using real time RT-PCR. Ct-values ranged between 21.09–23.48 in the wells where the virus replicated. The 50% tissue culture infective dose per milliliter (TCID50/ml) was calculated using the Reed & Muench method [36].

The virulent RVF 56/74 strain (passages history [37] and one passage in C6/36 cells) was propagated in BHK-21 cells. The virus was titrated in Vero cells and cytopathic effect was observed. The 50% tissue culture infective dose per milliliter (TCID50/ml) was also calculated using the Reed & Muench method [36].

### CxFV infection in mosquitoes orally exposed

Fourteen-day-old *Cx. pipiens* females were exposed for 60 min to CxFV infected blood (1:2) at 4 log_10_ TCID_50_/ml using the Hemotek feeder system. At 0, 3, 5, 7 and 10 days post-exposure (dpe), six fed females were harvested and frozen until analysis.

### CxFV intrathoracic inoculation in mosquitoes

A group of 36 *Cx. pipiens* females, 2–3 days of age, were intrathoracically inoculated with CxFV at 4 log_10_TCID_50_/ml diluted 1:2 in Dulbecco’s modified Eagle medium (DMEM). To study virus replication kinetics, these females were examined at 0, 3, 5, 7, 9 and 11 days post-inoculation (dpi). Bodies were analyzed from the 36 mosquitoes and saliva was harvested from all mosquitoes except from those corresponding to 0 dpi. Saliva was collected using a capillary technique as previously described [29]. As an inoculation control, a group of mosquitoes was inoculated with only DMEM.

### RVFV vector competence assay

The ability of RVFV to infect, disseminate and be transmitted by *Cx. pipiens* infected and non-infected with CxFV was evaluated by: infection rate (IR), disseminated infection rate (DIR), transmission rate (TR) and transmission efficiency (TE). IR refers to the proportion of mosquitoes with infected body among tested mosquitoes. DIR corresponds to the proportion of mosquitoes with infected legs/wings among the previously detected infected mosquitoes (i.e. body positive). TR represents the proportion of mosquitoes with infected saliva among mosquitoes with disseminated infection. TE represents the proportion of mosquitoes with infected saliva among the total number of mosquitoes tested [38].

Seven- to nine-day-old female mosquitoes that had never been blood-fed were used. Mosquitoes were reared and fed as previously described [29]. *Culex pipiens* intrathoracically inoculated with CxFV or with DMEM were tested for vector competence (VC) using a RVFV viral dose of 7.23 log_10_TCID_50_/ml. After the blood-feeding, CO_2_ was used to anesthetize the mosquitoes and fully-engorged females (FEF) were selected. The blood doped with RVFV was titrated in Vero cells. Ten percent of the specimens from each group were sacrificed and analyzed as a control of the inoculum. The rest of the mosquitoes were individually placed to cardboard cages (Watkins & Doncaster, Leominster, UK).

FEF were fed with sucrose (10%) *ad libitum* using soaked cotton pledgets. The presence of viral RNA in saliva was evaluated using two different approaches: FTA^TM^ cards (GE Healthcare, Little Chalfont, UK) soaked with Manuka honey (Manuka Health New Zealand, Te Awamutu, New Zealand) and a blue alimentary colorant, at 4 and 14 dpe and the direct extraction of mosquitoes’ saliva by capillarity at 14 dpe. At 4 and 14 dpe the FTA cards were left 24 h on the top of the mesh screen of all cardboard cages to allow the mosquito to feed on it. After FTA cards collection, they were resuspended in 0.3 ml of PBS and stored at -80 °C until tested. At 14 dpe, every mosquito was anesthetized with CO_2_ and dissected, and samples (legs/wings and bodies) were collected as previously described [29]. One hundred-fifty microliters from the saliva sample contained in DMEM medium were used for viral RNA extraction and the remaining 50 μl were used for RVFV isolation in a Vero cells monolayer. Cells were incubated for 7 days (37 °C, 5% CO_2_) and the cytophatic effect was evaluated.

### Virus detection

CxFV detection was performed using the real time RT-PCR protocol described by Bolling et al. [19] with minor modifications. The primer CxFV-F was modified as follows: 5'-CTA CGC TCT TAA CAC AGT GA-3' and RT-qPCR was carried out using Quantitec SyBr Green RT-PCR kit (Qiagen, Hilden, Germany). Samples were amplified using a 7500 Fast Real-Time PCR System (Applied Biosystems, Foster City, CA, USA) programmed as follows: 50 °C for 10 min, 95 °C for 10 min, 45 cycles at 95 °C for 15 s and at 57 °C for 35 s. RVFV RNA was extracted and detected as previously described [29].

### Statistical analysis

The frequency with which CxFV (+) and CxFV (-) mosquitoes get infected, disseminate, and transmit RVFV was compared by Fisher’s exact test. Ct-values in mosquito bodies, legs/wings and saliva 14 dpe were compared between CxFV (+) and CxFV (-) groups by a non-parametric Mann-Whitney test as data were not normally distributed. Differences in Ct-values in CxFV inoculated mosquitoes among dpi were assessed by means of a multiple comparisons Kruskal-Wallis test. *P*-values < 0.05 were considered statistically significant.

## Results

### CxFV replication kinetics in orally exposed *Cx. pipiens*

**Fig. 1:**
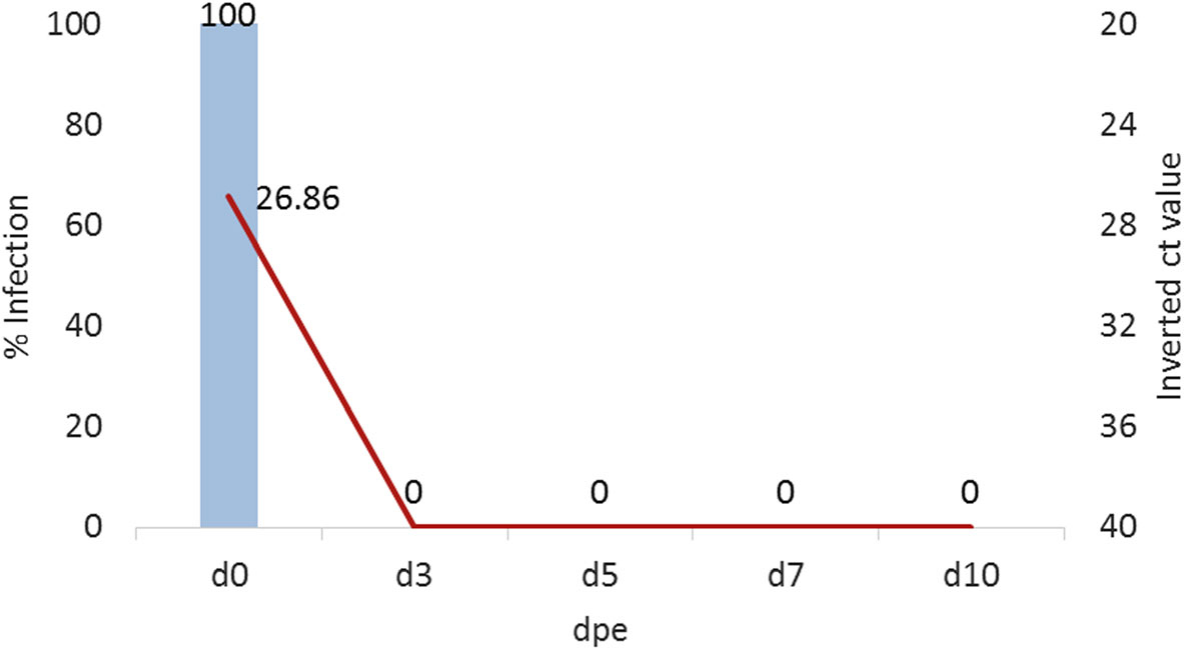
CxFV replication kinetics in *Cx. pipiens* oral infection. *Cx. pipiens* mosquitoes were not susceptible to CxFV infection following oral exposure. Columns show infection percentages and the line represents the Ct-values obtained by RT-qPCR. *Abbreviation*: dpe, days post-exposure

No CxFV replication was detected in *Cx. pipiens* exposed orally, suggesting that *Cx. pipiens* mosquitoes are not susceptible to CxFV infection by oral exposure. Although no positive CxFV was recorded in any tested female mosquito on 3, 5, 7 and 10 dpe, CxFV could be detected in all mosquito samples collected on 0 dpe, demonstrating that all mosquitoes were exposed to the virus (Fig. 1).

### CxFV replication kinetics in *Cx. pipiens* intrathoracically inoculated

**Fig. 2:**
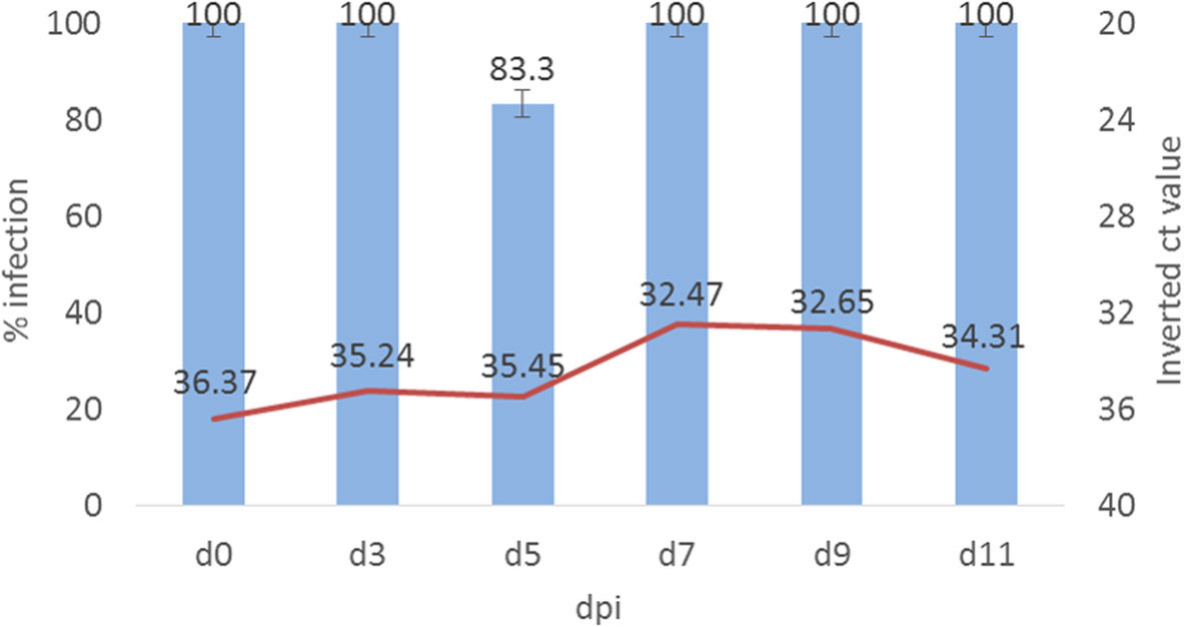
CxFV replication kinetics in *Cx. pipiens* intrathoracilally inoculated. *Cx. pipiens* mosquitoes were susceptible to CxFV infection after intrathoracic inoculation. Columns show infection percentages and the line represents the Ct-values obtained by RT-qPCR. *Abbreviation*: dpi, days post-inoculation

*Culex pipiens* intrathoracically inoculated with CxFV showed viral replication. Results demonstrated a high percentage of CxFV infection detected at all time-points analyzed. The obtained Ct-values were high, indicating low viral load. However, the multiple comparison Kruskall-Wallis test detected significant differences in viral loads among dpi (*H* = 16.692, *df* = 5, *P* = 0.005). The multiple comparisons of mean ranks indicated that the viral load in bodies of females tested at 7and 9 dpi was significantly higher than at 0 dpi (*z* = 3.33, *P* = 0.012 and *z* = 3.06, *P* = 0.033, respectively), showing CxFV replication within *Cx. pipiens* after intrathoracic inoculation (Fig. 2). All saliva samples tested at different time points were negative to CxFV.

### CxFV replication kinetics in *Cx. pipiens* co-infected with RVFV

**Fig. 3:**
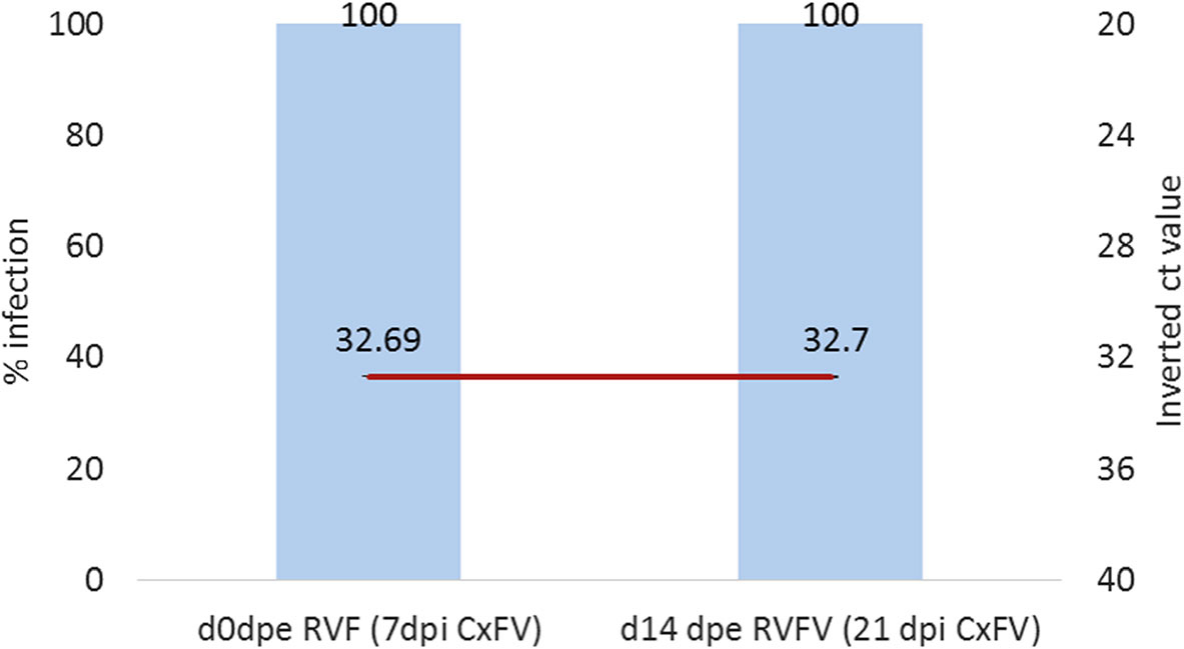
CxFV replication kinetics in co-infection with RVFV in *Cx. pipiens*. CxFV persisted after 21 dpi and was not influenced by RVFV exposure. Columns show infection percentages and the line represents the Ct-values obtained by RT-qPCR

CxFV replication was not affected by RVFV exposure in female *Cx pipiens* mosquitoes. Results showed that 21 days after CxFV inoculation and 14 days after RVFV exposure (14 dpe), bodies of all tested females remained positive to CxFV without significant differences (Fig. 3).

### RVFV infection, dissemination and transmission in *Cx. pipiens* infected and non-infected with CxFV

**Fig. 4:**
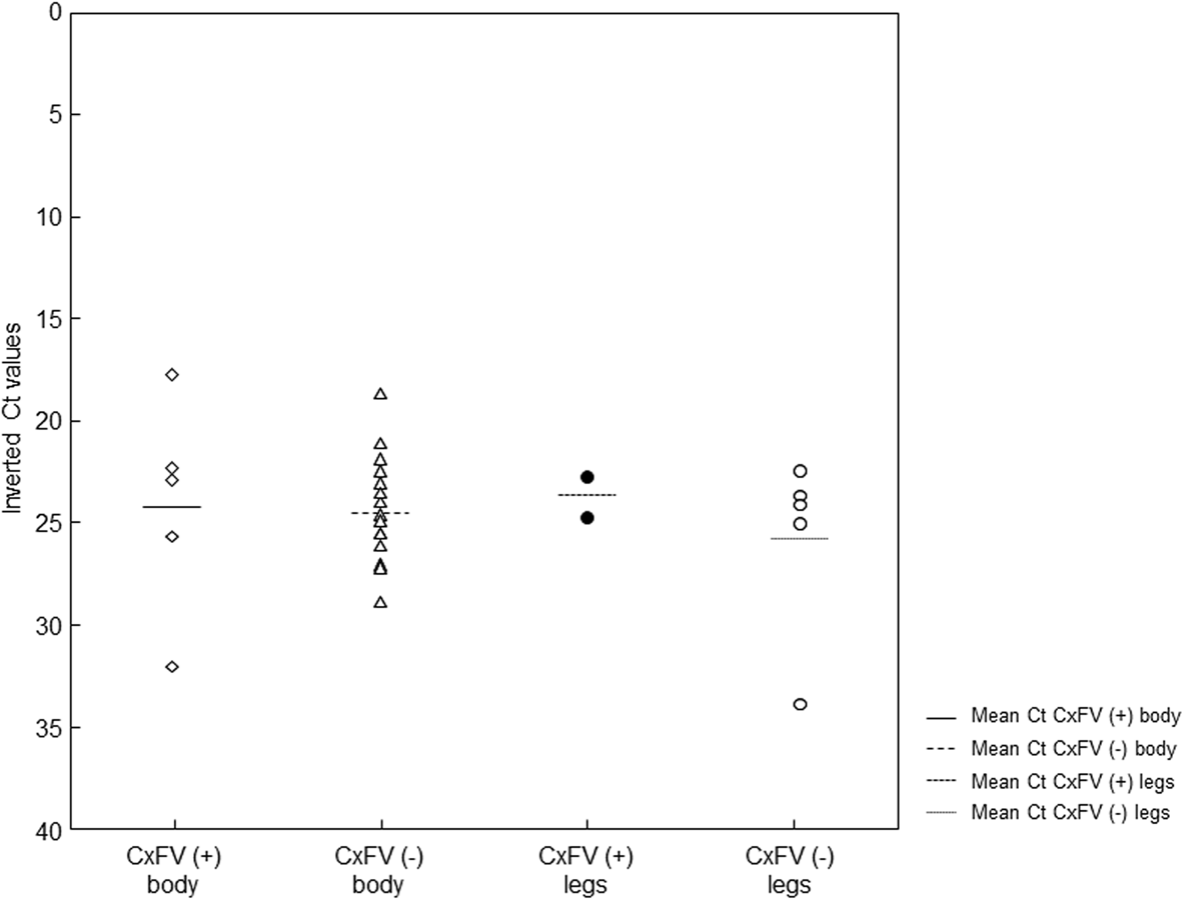
RVFV Ct-values in female mosquito bodies and legs infected and non-infected with CxFV. RVFV loads in female mosquito bodies and legs/wings were not affected by CxFV infection

Mosquitoes infected with CxFV and exposed to RVFV (*n* = 10; *n* = 1 hybrid form and *n* = 9 molestus form) and mosquitoes non-infected with CxFV and exposed to RVFV (*n* = 22; *n* = 5 hybrid form and *n* = 17 molestus form) were analyzed at 14 dpe. The percentages of RVFV infection, dissemination and transmission in analyzed mosquito females were not significantly different between females infected and non-infected with CxFV (Table 1). Moreover, RVFV loads in bodies and legs/wings were not significantly different between females infected and non-infected with CxFV (Fig. 4).

**Table 1:** RVFV infection, dissemination and transmission in *Cx. pipiens* infected and non-infected with CxFV

CxFV infection	IRg	DR	TR	TE
*+*	5/10 (50%)	2/5 (40%)	1/2 (50%)	1/10 (10%)
*-*	15/22 (68%)	5/15 (33%)	4/5 (80%)	4/22 (18%)

*Abbreviations*: *IR* infection rate, *DR* disseminated infection rate, *TR* transmission rate, *TE* transmission efficiency

All RVFV-positive saliva were detected in females with disseminated infection at 14 dpe. The Ct-values in mosquito saliva did not differ significantly between both groups, infected and non-infected with CxFV (Table 2). In addition, RVFV was detected in bodies, legs/wings or saliva of mosquitoes with (*n* = 27) and without (*n* = 5) *Wolbachia* (Table 2).

**Table 2:** Presence of RVFV in different samples of mosquitoes with positive saliva at 14 dpe. Ct-values of positive samples analysed by RT-qPCR are reported

Individuals	Legs and wings	Saliva	Saliva (CPE)	CxFV	*Wolbachia*
*Cx. pipiens molestus*	22.76	32.40	-	29.49	-
*Cx. pipiens molestus*	22.43	30.55	-	-	+
*Cx. pipiens molestus*	23.70	34.13	-	-	+
*Cx. pipiens molestus*	24.10	32.54	-	-	+
*Cx. pipiens molestus*	25.00	38.39	-	-	+

*Abbreviations*: -, negative; +, positive; *CPE* cytopathic effect

Regarding the forms of individuals from the *Cx. pipiens* hybrid colony, RVFV was detected in mosquito bodies, legs/wings and saliva of *Cx. pipiens* form molestus and in mosquito bodies of the hybrid form (Table 3).

**Table 3:** RVFV infection, dissemination and transmission in *Cx. pipiens molestus* form individuals, hybrid form individuals and all individuals of total mosquitoes tested

*Cx. pipiens*	IR	DR	TR	TE
Molestus form (individuals)	16/26 (61%)	7/16 (44%)	5/7 (71%)	5/26 (19%)
Hybrid form (individuals)	4/6 (67%)	0/4 (0%)	–	0/6 (0%)
Total (colony)	20/32 (62%)	7/20 (35%)	5/7 (71%)	5/32 (16%)

*Abbreviations*: *IR* infection rate, *DR* disseminated infection rate, *TR* transmission rate, *TE* transmission efficiency

## Discussion

The isolation, identification and characterization of numerous insect-specific viruses in recent years are of particular interest. They can coexist with pathogenic arboviruses in mosquito populations and may potentially affect the transmission of vector-borne infectious diseases. While there is extensive genetic and phenotypic characterization of insect-specific flaviviruses, little is known about the interactions between them and their mosquito hosts and other arboviruses and the potential public health significance of these associations [39]. Relatively few studies have been performed on co-infections with other flaviviruses such as WNV [18–20]. To the best of our knowledge, the present study is the first to perform a co-infection with two viruses from different genera, CxFV (*Flavivirus*) and RVFV (*Phlebovirus*).

The mechanism through which natural mosquito populations become infected with CxFV is not yet well defined. Our results strongly suggest that *Cx. pipiens* females are not susceptible to CxFV upon oral exposure. This is in agreement with previous studies showing transmission of insect-specific viruses solely among their invertebrate hosts by vertical route [1, 5]. Intrathoracic inoculation of CxFV in our study, however, indicates that the virus may have the potential to replicate in *Cx. pipiens* females at least for 21 days, establishing a possible CxFV persistent infection. Nevertheless, CxFV could not be detected in saliva after 14 dpi. Our results are in line with a previous report by Kent et al. [20] who showed that CxFV Izabal intrathoracically inoculated to *Cx. quinquefasciatus* females was not found in the saliva.

Vector competence for RVFV was examined at 14 dpe in one *Cx. pipiens* colony artificially infected with CxFV by intrathoracic inoculation. The percentage of mosquito females that became infected, developed a disseminated infection, and transmitted RVFV was not significantly different between females infected and non-infected with CxFV. We assume that CxFV may have co-evolved with their mosquito host evading their immune system without affecting its function against a subsequently-infecting virus. As such, the molecular mechanisms that allow co-existence of both CxFV and RVFV are not well defined and need more extensive studies. Furthermore, RVFV RNA levels observed were also not significantly different suggesting that CxFV does not affect RVFV replication. This is in agreement with other published studies where co-infection of CxFv and WNV has been performed. Similarly, Kent et al. [20] investigated the vector competence for WNV of *Cx. quinquefasciatus* mosquitoes intrathoracically inoculated with CxFV Izabal, and also observed no significant differences in WNV titers between CxFV-positive and CxFV-negative mosquitoes at 14 dpi. Another study that tested the vector competence for WNV in two *Cx. pipiens* colonies [19], one colony CxFV naturally infected and the other CxFV non-infected, reported no significant differences in WNV dissemination between both colonies at 14 dpe. However, significant differences were observed at 7 dpe, being significantly higher in the CxFV-negative colony than in CxFV-positive colony. These results suggested a competitive interaction between CxFV and WNV indicating a possible early suppression of WNV replication by CxFV infection in *Cx. pipiens*. Vector competence is influenced by the time-point examined and by genetic differences between mosquito populations [40] as well as genetic diversity and fitness of a laboratory colonized population [41, 42]. All these factors must be taken into account for co-infection studies in mosquitoes.

The *Cx. pipiens* colony used in the present study was naturally infected by *Wolbachia* spp. This may have influenced the vector competence of infected mosquitoes as shown in a previous study [43]. Our results showed that RVFV was detected in bodies, legs/wings or saliva of mosquitoes with (*n* = 27) and without (*n* = 5) *Wolbachia.* Due to the small sample size, further studies regarding this issue are needed to explain the potential interference of *Wolbachia* in arbovirus-vector interactions.

The present study and our previous report [29] allow us to assure that the *Cx. pipiens* hybrid colony of Gavà can become infected, disseminate and transmit RVFV. The IR and DIR obtained were lower than those reported by Turrell et al. [44] when a *Cx. pipiens* hybrid colony was exposed to a similar RVFV viral dose (10^7.5^ PFU/ml) at 14 dpe. Regarding the forms of *Culex pipiens*, RVFV was detected in mosquito bodies, legs/wings and saliva of *Cx. pipiens* form molestus (*n* = 26 tested). Thus, our findings in the present work also showed that the individuals of molestus form within the hybrid colony disseminated and transmitted RVFV. However the virus was only detected in mosquito bodies in hybrid form (*n* = 6). These results may suggest that the individual form might determine the RVFV dissemination and later transmission, suggesting a strong midgut barrier in hybrid form in *Cx. pipiens* individuals.

The insect’s immune responses largely determine the viral load, extrinsic incubation period, and mortality of the insect vector after viral infection, all of which directly affect the outcome of disease transmission [45, 46]. Exposure to one microorganism can provide cross-protection against another microorganism. Specific examples of the super-infection exclusion hypothesis based on the idea of homologous interference, which is the ability of an established infection with one virus to interfere with secondary viral infection, has been documented in cell culture not only with flaviviruses [47–50], but also with other arboviruses of the genera *Alphavirus* [51], *Orbivirus* [52] and *Vesiculovirus* [53, 54]. The study of Bolling et al. [19] reported that CxFV could alter the WNV infection on mosquitoes although it did not exclude WNV infection. However, a positive correlation between WNV and CxFV infection of field-collected *Cx. pipiens* mosquitoes from Illinois has been observed, suggesting that there could be a biological suppression that mediates an increasing susceptibility to naturally WNV infected mosquitoes [56]. Moreover, WNV transmission was enhanced in the Honduras colony when mosquitoes were inoculated simultaneously with WNV and CxFV Izabal [20]. To our knowledge, nothing was known about the potential interference of CxFV in the mosquito infection by other arboviruses not belonging to *Flavivirus* genus. Our results, for the first time, indicate that CxFV infection in *Cx. pipiens* might not alter the immune system to interfere with the RVFV infection in case of RVFV introduction in *Cx. pipiens* populations.

## Conclusions

This is the first study to assess the potential interference of an ISF on RVFV transmission. We have shown that CxFV does not affect RVFV infection, dissemination and transmission. Mosquitoes persistently infected at the assessed conditions may not be used as a preventive intervention strategy for blocking the transmission of RVFV. Further studies using mosquitoes naturally infected with CxFV should be performed to deepen the knowledge in the natural CxFV infection and to elucidate consistent trends for RVFV vector competence in CxFV artificially and naturally infected *Cx. pipiens* populations. Altogether, it is necessary to highlight the importance of deepening the knowledge on the interaction of ISF circulating in mosquito populations present in an area where the potential pathogenic arboviruses can be introduced in order to better assess arbovirus risk transmission. Examining associations between insect-specific viruses such as CxFV and RVFV and other arboviruses important for human and animal health will provide significant new insights into both arbovirus biology and public health.

## Abbreviations

CxFV: Culex flavivirus
DIR: Disseminated infection rate
DMEM: Dulbecco’s modified Eagle medium
FEF: Fully-engorged females
IR: Infection rate
ISFV: Insect-specific flaviviruses
MBFV: Mosquito-borne flaviviruses
RVFV: Rift Valley fever phlebovirus
TE: Transmission efficiency
TR: Transmission rate
WNV: West Nile virus
